# Complexity synchronization in emergent intelligence

**DOI:** 10.1038/s41598-024-57384-5

**Published:** 2024-03-21

**Authors:** Korosh Mahmoodi, Scott E. Kerick, Piotr J. Franaszczuk, Thomas D. Parsons, Paolo Grigolini, Bruce J. West

**Affiliations:** 1 US Combat Capabilities Command, Army Research Laboratory, Aberdeen Proving Ground, MD 21005 USA; 2 Department of Neurology, Johns Hopkins University School of Medicine, Baltimore, MD 21287 USA; 3 Computational Neuropsychology Simulation Laboratory, Edson College, Arizona State University, Tempe, AZ 85004 USA; 4 Center for Nonlinear Science, University of North Texas, Denton, TX 76203 USA; 5 Office of Research and Innovation, North Carolina State University, Raleigh, NC 27695 USA

**Keywords:** Complexity synchronization, Modified diffusion entropy analysis, Emergent intelligence, Adaptive environment, Multi-agent-based-modeling, Selfish algorithm, Reinforcement learning, Complex networks, Nonlinear phenomena, Statistical physics

## Abstract

In this work, we use a simple multi-agent-based-model (MABM) of a social network, implementing selfish algorithm (SA) agents, to create an adaptive environment and show, using a modified diffusion entropy analysis (DEA), that the mutual-adaptive interaction between the parts of such a network manifests complexity synchronization (CS). CS has been shown to exist by processing simultaneously measured time series from among organ-networks (ONs) of the brain (neurophysiology), lungs (respiration), and heart (cardiovascular reactivity) and to be explained theoretically as a synchronization of the multifractal dimension (MFD) scaling parameters characterizing each time series. Herein, we find the same kind of CS in the emergent intelligence of groups formed in a self-organized social interaction without macroscopic control but with biased self-interest between two groups of agents playing an anti-coordination game. This computational result strongly suggests the existence of the same CS in real-world social phenomena and in human–machine interactions as that found empirically in ONs.

## Introduction

In three recent papers^1–3^ the authors have used the scaling behavior of dynamically generated time series to hypothesize the existence of a new form of synchronization having to do with the optimally efficient exchange of information between and among organ-networks (ONs) within the human body which is itself a network-of-ONs (NoONs). This new form of synchronization is called complexity synchronization (CS). Unlike traditional forms of synchronization having to do with the emergence of coherent collective actions among dynamic entities ranging from fireflies to people and engagingly described by one of the field’s leaders^4^, this addition of CS to the new science involves the synchronization of the scaling indices of time series rather than the correlation-like character of the central moments.

Normal synchronization is the entrainment of emergent dynamic global variables of two or more interacting networks including the critical dynamics of many-body phase transitions in the taxonomy of the expanding definition of synchronization^5^. The global variable of these complex networks is given by a discrete time series which is typically renewal^6^ with an inverse power law (IPL) for the time intervals (τ) between events in the waiting-time probability density function (PDF) (1/τ^µ^) and the IPL index *µ* is equal to the fractal dimension of the time series *D*^7^. The measure of complexity of a fractal time series is the fractal dimension which when the network interacts with another network of comparable complexity the complexity measure changes over time becoming the multifractal dimension (MFD) *D*(*t*). The MFD is the measure of each network’s complexity in a network-of-complex-networks. It has been determined by processing the simultaneous datasets consisting of the cardiovascular, the respiratory and the electroencephalogram time series that their MFDs are in synchrony which is to say this phenomenon is given by CS^1–3^.

We now have a definition of CS but what it entails is another matter altogether and the details of those entailments, necessary to understand the calculations done here are presented in detail in section *Basic concepts and models*. Herein we explore the potential connection between CS phenomena recently observed in the interaction among the time series generated by human heart, lungs and brain^1,3^ with that of phenomena generated by the interaction of correspondingly relative complexity of social Selfish Algorithm agents (SA-agents) of^8^. Note that the detailed dynamics of the two kinds of networks, the first one being the interaction of three human organ-networks (ONs), whereas the second is the surrogate interaction of an arbitrary number of SA-agents, can be very different yet we show that the multifractal dimensional (MFD) nature of the scaling indices of their separate and distinct time series satisfies the conditions for the same kind of CS. The equivalence of the information content of the human physiological and a surrogate model of social networks is established dynamically by the direct calculation of the collective behavior of the SA-agents. The details of the model used in the calculations are given in the *Methods* section, but a general overview of the calculations is useful to avoid becoming lost in the weeds.

### Overview

This CS of the scaling indices of the SA-agent interacting groups strongly suggests that above the level of time series synchronization^5,9^ there is a higher-order synchronization of the time-dependent scaling indices^3^. This higher-order synchronization can arise even in the absence of cross-correlations of the central moments of the underlying time series, such as in their cross-correlation function. But to see this behavior clearly requires laying the foundation for the growth and formation of a network from the mutual interactions of a collection of SA-agents, bearing in mind that the dynamics of such self-serving (basically selfish) agents has in the past led to the counter-intuitive result that the social network that is so formed derives optimum value from their interactions^8^.

Herein we do not initiate the interaction on a lattice, but instead allow the network’s structure to emerge as part of the calculation. Starting the SA-agents from random positions and moving in random directions on a two-dimensional lattice with periodic boundary conditions, thereby walking on the two-dimensional surface of a three-dimensional torus in phase space, the SA-agents, each trying to selfishly increase their own payoff, learn to form multiple large collectives, such as swarms and other such collectives.

Each SA-agent learns to modify its model of the world to anticipate/engineer its dynamic environment (i.e., the behavior of other SA-agents), whose purpose is to increase its own payoff. In this model, to make decisions (i.e., in which direction to move), the SA-agent first adaptively decides to either rely on the information from neighboring SA-agents of the in-group or out-group neighboring SA-agents (using a reliance propensity/probability). In the first case, the SA-agent picks its next direction to move as the average of those in-group agents. Otherwise, using the information from the neighboring out-group members of the other SA-agent group, the SA-agent anticipates their average position and adaptively decides what its next direction to move will be, either away from or towards that position.

Although the bulk of the discussion presented herein is in terms of the numerical simulation of social interactions among selfish individuals focusing on the resulting emergence of group intelligence, we do not want to leave the impression that our remarks are restricted to such applications. For example, if wealth is a measure of social value, so that in reaching their goal of individual wealth maximization, the SA-agents incidentally and counter-intuitively also optimize the wealth of the social group^10^. In their discussion of empirical paradox resolutions, they^10^ show that an analogous argument applies to collections of other social animals as well as to physiologic network of organ-networks (NoONs).

#### Kinds of intellect

The intelligent behavior of animal collectives is one research focus of Couzin^11^, who, using an integrated approach involving both fieldwork and laboratory experiments guided by mathematical models and computer simulations, attained unparalleled understanding of animal group behavior. For example, in his remarkable work with army ants he unveiled how such ants form traffic lanes in their movement that is optimum for the avoidance of congestion. The social value in this latter case being the efficiency of movement to carry out a group task.

The intelligence of our multi-agent-based-model (MABM) society is emergent since there are no macroscopic control parameters, such as noise or temperature. The first is often used in swarm intelligence models^12^, and the latter is often used in the social application of the Ising model of phase transitions in which the social model has the control ’temperature’ being interpreted as the level of excitation of the social network^13^. In the present MABM society, the adaptive environment of each individual SA-agent replaces the role of the macroscopic control parameter. Proceeding in this vein, the ensemble average of reliance propensity, oppose-follow propensity, and the order parameter time series of two SA-agent-groups represent the dynamics of the mutual adaptive interactions in our surrogate social network.

#### Diffusion entropy analysis (DEA)

Consider the electrocardiogram (ECG) measurement (continuous trace) of an individual’s heart activity. This continuous curve is replaced by a point process wherein an event is defined by the peak of the ECG and the health of the individual’s heart is determined by the statistics of the time interval between these events referred to in the literature as the heart rate variability (HRV). If $\xi$(*t*) is the discrete HRV time series and it scales in such a way that given the constant *λ* we obtain the homogeneous scaling function $\xi (\lambda t) = \lambda^{\delta } \xi (t)$ enabling us to measure the time series complexity by means of the diffusion entropy analysis (DEA):

1 $$dY\left( t \right)/dt = \xi \left( t \right)$$

In this way the HRV statistics are determined by the ensemble of trajectories given by the diffusion process *Y* (*t*) defined by the simplest of rate equations.

We can introduce a moving window of size *l* to generate a PDF *P*(*y*, *l*) by sweeping the window through the time series and constructing an ensemble of histograms from the resulting trajectories. The PDF for the diffusion process is then inserted into the Wiener-Shannon (WS) information entropy in this window to obtain:

2 $$S\left( l \right) = - \smallint dyP\left( {y, l} \right)log_{10} P\left( {y, l} \right)$$

The scaling of the dynamic variable $\xi$(*t*) from the diffusion process transfers to the scaling form of the PDF^14^:

3 $$P\left( {y,l} \right) = F(y/t^{\delta } )/t^{\delta }$$

where *δ* is the scaling index and *F*(*.*) is an unknown analytic function of its argument. Inserting the scaling PDF into the WS-entropy yields for the entropy relative to a constant reference state *S*_0_:

4 $$\Delta S\left( l \right) = S\left( l \right) - S_{0} = \delta log_{{{1}0}} \left( l \right)$$

This result is the heart of the technique because if DEA processing of empirical data and graphing the entropy versus *log*_10_(*l*) yields a straight line the slope of that line is the scaling index *δ* of the PDF.

The DEA reveals that when the scaling indices *δ* ’s of these time series are time-dependent and in synchrony with one another, meaning their statistics change over time relative to their changing environment^2,3^ implying that each scaling index is related to a specific MFD and the CS is expressed in terms of the synchrony of these MFDs. We demonstrate in the “Results” Section that although the cross-correlation between these time series does not reveal the synchronous interrelations among the groups, there is strong cross-correlation between their MFD time series. This cross-correlation is a CS providing a reasonable working measure of the intelligence of the overall network.

### Basic concepts and models

To generate CS in a social context, we model the competition between two groups of SA-agents. Then, using a modified DEA data processing technique, we study the high-order cross-correlation between the multifractal time series of the scaling indices characterizing different mean fields emerging from the distinct dynamics of the two groups. Put more simply, the two time series, one for each group of SA-agents are processed to determine the behavior of their separate and distinct scaling indices *δ* in time which we have shown elsewhere^3,15^ also determines the MFDs of the separate time series.

### Self-organized temporal criticality (SOTC)

The depth of our understanding of synchrony stands in sharp contrast to the shallowness of our collective insight into the mystery that is complexity whose problems have gained preeminence in science over the last decade paralleling the sharp rise in the application of Network Science to our understanding of how collective behavior emerges from critical dynamics. One might say that the modern view of complexity began with the acknowledgment that a new branch of science had formed around the behavior of a many-degree-of-freedom system governed by the emergent dynamics of spontaneous self-organized criticality (SOC)^16^. Moreover, both bottom-up resilience and top-down vulnerability arise from emergent processes captured by spontaneous self-organized temporal criticality (SOTC)^17^. The term criticality is used to denote the dynamic condition corresponding to the onset of phase transitions, generated by the adoption of suitable values of control parameters, whether the parameter values are externally controlled or self-induced by the internal dynamics.

#### Temporal complexity

The systemic environment of a complex network is comprised of other complex networks, which creates a network-of-networks (NoNs), that is either competing or cooperating. As such, for a NoNs to carry out a specific task, each interacting network should be mutually adaptive to its environmental changes. For example, the environment of a particular ON consists of the other adaptive ONs within a NoONs. It has been shown that the time series generated by the internal dynamics of these separate ONs, e.g., two such ONs in the human body generate the respiration and cardiac datasets, both of which have temporal complexity^18,19^, meaning that their statistics are governed by MFD time series given by Crucial Events (CEs).

CEs are defined by discrete time series consisting of events with time intervals τ between consecutive events that are statistically independent of one another. The defining properties of renewal events (RE) were put together by William Feller^20^ wherein he developed the mathematical foundation of RE theory. CEs are therefore REs, however, although CEs are renewal they have IPL PDFs. The IPL PDF of the time intervals, τ’s, between sequential events is *ψ* (τ) ∝ τ^−µ^ and the IPL index *µ* is in the domain 1 < *µ* < 3, or equivalently, such CE time series have inverse power law spectra of the form *S*(*f*) ∝ *f*^−*β*^ where 0 < *β* = 3 − *µ* < 2^21^.

The utility of REs have been found in modeling the firing of neurons^22^, to analyze the reliability and maintenance of networks modeled generally with time-dependent parameters, these and many more are considered in the brilliant work of Cox^23^ and which were applied to medical phenomena in^14^. For most physiological data 2 < *µ* < 3, with *µ* ≃ 2 in a healthy brain^18^. To unambiguously measure the IPL index *µ* of a single complex trajectory, a modification of DEA was developed^24^, mainly because in the ergodic region of 2 < *µ* < 3 the second moment does not exist, and in the non-ergodic region of 1 < *µ* < 2 both the first and second moment do not exist, therefore, complexity analysis based on the second moment, such as detrended fluctuation analysis (DFA), can give misleading results when applied to such datasets as shown in the “Discussion” section.

A modified DEA applied to a fractal time series provides a scaling index *δ* ∈ (0*,* 1], with *δ* = 0*.*5 corresponding to Brownian motion. The scaling index *δ* is connected to the temporal complexity IPL index *µ* of an ergodic signal by *µ* = 1 + 1*/δ*. In the present work, we show that the scaling index *δ* found in mutual adaptive networks is time-dependent, and those in NoNs are in synchrony with one another. For more details on ways to modify DEA, see “Methods” section and^1,3^.

#### CS differs from CM

In^1,3^, we showed that the scaling of simultaneously recorded time series from physiological ONs, e.g., the brain, heart, and lungs, obtained by a modified DEA processing produces distinct MFDs in synchrony with one another designated as CS. Herein we study the occurrence of surrogate phenomenon using surrogate datasets generated from interacting SA-agents to show that CS is a general emergent property of mutually adaptive environments, whether using physiological datasets or model-generated surrogate data. The CS between SA-agents having the same mathematical infrastructure as that of physiological ONs suggests the hypothesis that SA-agents could be used as trainers, rehabilitators, decision-makers, etc., to form successful human-EI hybrid teams^25^. Recent studies have shown that individuals can recuperate more rapidly from surgery and other invasive procedures intended to correct the negative effects of disease or injury through the use of life support systems that operate at the body’s natural bio-frequencies. The same observation has been clinically shown to reverse the degenerative effects of neurodegenerative diseases such as Parkinson’s and Alzheimer’s Disease. Crucial Event Rehabilitation Therapy (CERT) describes medicine as the operational control of the functions of the human body treated as a network-of-networks, with 1/f-variable crucial events coding the dynamic states of health and disease through information flow within a network and information exchange between biomedical networks^25^.

Complexity matching (CM)^26^, on the other hand, identifies the transfer of information as being maximal when the time- dependent complexity level identified by the MFDs of two interacting complex networks are the same^27^. It is a phenomenon that has been fully explained only by means of the Shannon-Wigner information entropy, i.e., the complexity of the information-rich network does not change when interacting with an information-deficient complex network, however, the information-poor network slowly increases in (information) complexity until it is on a par with the information-rich network. The exchange of information does not follow the energy gradient and apparently differs from the traditional form of the Second Law of Thermodynamics.

When two physical networks of approximately the same size are brought into contact with one another, the hotter network loses energy to the cooler network until they reach thermal equilibrium and the temperature is uniform across the two. Information transfer does not work this way. It is true that the information-rich network increases the complexity level of the information-poor network through the transfer of information, doing so without necessarily losing any of its own complexity, and therefore its information content remains the same. This kind of implementation of CM has been used to interpret the arm-in-am walking of elders who have impaired gate patterns with younger persons to reinstate a better gait pattern^28^. Moreover, it suggests that complexity is a qualitative property of an ON, however, its measure, the MFD, is quantitative.

By way of contrast, in CS when two or more networks strongly interact, their complexities change synchronously. Consequently, CM cannot by itself explain the CS phenomenon. In^15^, two SA-agents were shown to strongly interact to model the experimental results of rehabilitation in patients with gait disorder when they walked arm-in-arm with a care-giver^29^. This model can lead to a theoretical foundation for CS, showing that the network with lower complexity can improve its complexity through information obtained from the more complex network.

#### CS measures EI

Here, we point out that a complex network generating a CE time series is not necessarily intelligent. For example, blinking quantum dots, liquid crystals, earthquake waves, solar flares, etc., clearly, are not intelligent, while the time series associated with their dynamics have a complexity index *µ* which is close to that of biological networks such as the brain^30,31^. These processes host CEs time series that could be termed mechanical to distinguish them from the CEs time series generated by ONs, but which share the same statistical properties. On the other hand, intelligence is an emergent property, i.e., individuals contribute to realizing an achievement they could not achieve alone, and in return, the formed group’s intelligence steers an individuals’ behavior^8,32^. So, an EI network continuously adapts to its changing environment to better anticipate/engineer the next state of the environment. By attaining that next state, the organization obtains the advantage of being a step ahead of the decision-making in time and, consequently, improves its self-interest/survival. This means that in an EI network CEs emerge as a result of bi-directional interactions between its elements, and in addition the EI network is able to change its CEs statistics (scaling index) to that of its adaptive environment, a phenomenon that is quantified using CS. So, apparently, an EI time series can host intelligent CEs.

In this work, we use MABM to create EI, and by studying the dynamics of the emerging networks, we find the CS phenomenon to be in evidence, which was only recently uncovered in the analyses of simultaneously recorded brain, heart, and lung time series datasets^1,3^. This finding suggests, but does not guarantee, a universality of CS beyond the confines of physiology and sociology and to be a property resulting from the ’intelligent CEs’ that make up the respective time series.

### Emergent intelligence

The interactions between atoms, which are the building blocks of matter, are identical and non-adaptive. Contrariwise, the interactions between entities, such as individuals within a social network or a network of neurons within a brain, are non-identical and adaptive, resulting in emergent properties. So, to model such complex networks, we need building blocks that can update their interactions based on their internal state. Agents have been used for this purpose because they can receive information about their changing environment, process that information, make decisions based on the application of weighted valuations, and implement those decisions to manipulate their environment (such as sending true or deceptive information, moving, etc.).

#### Mutual adaptive environment breeds intelligence

To the best of our knowledge, we^17,32^ were the first to use mutual-adaptive agents in an adaptive environment wherein each agent *i*, located on a 2D lattice, was able to choose the microscopic control parameter value (*K*_*i*_) reflecting the extent to which it would imitate the decision of its neighboring agents. The agents could either cooperate (*C*) or defect (*D*). Based on the agent’s decision and the decision of its neighbors, payoffs were received according to the payoff matrix of a prisoner’s dilemma (PD) game. Each agent used an Ising-like rate function containing the parameter *K*_*i*_ to make its decision. At the end of each trial, the agent compared its current payoff with its previous payoff and used that difference as feedback to reinforce its *K*_*i*_ choice for the subsequent decision-making trial.

The idea of using the successive differences in payoffs as feedback and thereby bringing the network to criticality surfaced in^33,34^ wherein it is shown that the act of increasing the control parameter of the Ising-like rate towards its critical value has the remarkable effect of increasing the average payoff of the network. So, given the continuous weighting updates of the microscopic imitation parameter *K*_*i*_ of the agents, based on their payoff feedback, the entire network reaches criticality, this phenomenon has been identified as SOTC in^17,32^. SOTC results in a robust and resilient team of cooperators. It is important to note that SOTC is distinguished from its precursor self-organized criticality (SOC) in that SOTC does not include macroscopic control parameters (such as temperature as in the Ising model^35^ or noise as in^36^). Instead, the mutual interaction between the agent and its adaptive environment modifies its microscopic control parameter *K*_*i*_, and the network, as a whole, reaches a global understanding of the advantage of cooperation and long-range correlation. In other words, each agent, although acting in its own self-interest, contributes to the emerging network’s intelligence, and such intelligence controls the behavior of agents and the system as a whole.

#### What is different now?

Criticality as the antecedent to SOC has been hypothesized to be able to describe the dynamics of biological systems such as the brain^16^. Despite some success in using criticality to explain some aspects of intelligence; intelligence so defined has insurmountable limitations:

- The theory of criticality is typically controlled via a macroscopic control parameter rather than being self-controlled^16,37^.
- The network’s units are located on a predefined lattice rather than being on an initially free aggregation out of which a lattice of interacting units is dynamically formed, consequently a lattice emerges through the interactions of the units^38^.
- The network at criticality is highly responsive to weak perturbations and is consequently not robust^39^.
- The response of a network at criticality to a stimulus is prolonged. The process is known as *critical slowing down*^40^, meaning that its dissipation is weak.

While SOTC overcomes these limitations^17,32^, it still needs a predefined two-dimensional lattice network, even though the connectedness of the network on this lattice changes over time. Also, it uses the Ising-like rules for updating the decisions. Note that an agent in SOTC does not make its decision (*C* or *D*) independently of the other agents. Instead, it decides whether to increase or decrease the biased weighting of its microscopic imitation (*K*_*i*_) based on the decisions of its neighbors during the playing of PD.

Chialvo et al.^41,42^ suggested a long-range correlation, parameterized to the autocorrelation function, as a fundamental feature of intelligence and criticality. Using an autocorrelation function as feedback to update the macroscopic control parameter, they showed that the network can stay within a narrow region of criticality. Their work might be considered a top-down version of SOTC^17,32^. However, it implies the assumption that the NoNs somehow knows that long-range correlation (LRC) is beneficial, while in SOTC, the LRC is a byproduct of bottom-up self-organization rather than being the generator of LRC. Also, it is not always true that LRC corresponds to self-organization. It has been shown^43^ that if the LRC is the result of long-term memory (such as in Fractional Brownian Noise), it is a sign of the organization’s impending collapse rather than indicating a robust, self-organizing network at a high level of performance, as it would if generated by SOTC.

## Methods

In this section we develop the network computational techniques necessary to calculate how intelligence emerges from CS in a social setting. This form of EI is called swarm or group intelligence in anthropological literature.

### Selfish algorithm (SA)

To go beyond the limits of the SOTC agents in^8,37^ the selfish algorithm (SA) agent (SA-agent) is introduced to create a novel architecture for modeling emergent intelligence (EI), see^44,45^ for discussions of the variety of well-defined EIs. Here we interpret EI to be the pattern of decisions made by the collective of interacting SA-agents produces a greater reward for the social group than would be obtained for the same group but with each member making an independent decision. The EI observed herein arises from the SA-agents making their decisions based solely on reinforcement learning; an SA-agent assigns a *P* value for each choice and reinforces it according to the difference between its corresponding last two payoffs. In other words, an SA-agent biases its *P*s, using the feedback of its two last payoffs to anticipate/engineer the next behavior of the other SA-agents in its environment. That this will result in the suggested form of EI is not obvious and only reveals itself through the proper experimental computations.

SA-agents can make various decisions, such as *C* or *D* in the PD game, trust or not trust the decision of other SA-agents, interact or not interact with specific SA-agents, etc. The set of the *P*s of the SA-agents’ choices across events creates temporally complex networks (such as a network of trust and a network of connections) that emerge among the SA-agents because of their mutually adaptive interactions. These networks accumulate what SA-agents learn through the experience of playing with one another. Note that the intelligence emerges among the SA-agents without assuming a pre-existing network structure, and instead, SA-agents form multi-layer temporally complex networks from a lattice-free initial state^37^.

In^8^, SA-agents play the PD game with one another and have choices C or D. In playing the PD game, SA-agents reach a level of mutual cooperation over time, depending on the value of the temptation to cheat (*T*). Adding the choice of trusting or not trusting the decision of other SA-agents, and the choice of with whom to play, SA-agents show higher levels of mutual cooperation and robustness. Emergent networks (ENs) resulting from SA-agent interactions are resilient to perturbations and maintain high performance. In^8^, some SA-agents were replaced with zealots (i.e., SA-agents that only chose D) to show that the ENs maintain their functioning by isolating the zealots.

It is important to note that from time to time some SA-agents defect (to get a free ride) and receive the benefit of being in a pool of cooperative agents, but quickly, the other SA-agents react to such behavior. For example, these SA-agents will either not play with such SA-agents or will play as defectors with them. In this way, other SA-agents force the defecting SA-agents to return to cooperative behavior (Fig. 2 in^8^) and thereby teach the deviant SA-agent how to be a ’good citizen’. Such bottom-up control indicates a healthy, robust, adaptive social group.

The main advantage these SA-agents possess is that the emergent temporally complex networks are interpretable since they are based on fuzzy logic (*P*s) and originate from self-interest. For example, the temporally complex network of connections surrounding the zealot demonstrates that SA-agents did, in fact, isolate the zealot (see Fig. 11 in^8^). In contrast, the networks formed from traditional artificial intelligence (AI) networks, such as deep neural networks, are far less interpretable as part of the theory and have been described as “black boxes”. Although the SA-agents in^8^ have three types of choices, it is easy to equalize their influence on the network dynamics by adding more choices, such as deception, for real-world applications.

### Payoffs as environmental feedback

In MABM, the payoffs of the game that SA-agents play give them feedback for their actions toward others, letting them update their weighted biases for future decisions toward optimized performance. We can consider the game as a metaphor for the fixed part of the environment in which SA-agents are co-located, such as obstacles they must overcome to succeed. On the other hand, the SA-agents’ behavior changes over time, creating an adaptive environment for one another as in the NoNs.

For example, to model an environment where only cooperation results in optimal, long-term success, it is common to set the SA-agents to play the PD game. The payoffs of the PD game are as follows: When the pair of SA-agents make the decision cooperation C, each gets 1. If one SA-agent decides C and the other decides *D*, they receive 0 and 1 + *T*, (*T* > 0 is the temptation to cheat), respectively. If both agents decide *D*, each receives 0. Note that 1 < 1 + *T*, so agents are tempted to choose *D* over *C*, and 1 + 1 > 1 + *T* + 0, which means cooperation is a better option in the long run.

SA-agents are incentivized to change their decisions based on the feedback of the PD game’s payoffs, and if they learn over time, they might find that mutual cooperation is the most beneficial decision. These payoffs can be representative of an environment where two SA-agents need to reach a super-ordinate goal, but they can only do so if one SA-agent elects to assist the other and shares the benefit (mutual *C*). But the SA-agent in the super-ordinate position may also decide to hold on to the benefit (*D*) or never choose to assist the other (mutual *D*). Over time, an SA-agent can change decisions (and so indirectly change the decisions of the other SA-agent) to reach the "eureka" moment of mutual cooperation.

### Selfish algorithm agent (SA-agent)

SA-agents are the building blocks of the EI studied herein. An SA-agent makes decisions based on the *P*s it assigns for different choices and updates them over time throughout the interaction with other SA-agents. For each SA-agent, other SA-agents act as mutual adaptations within environments, as defined previously using NoONs in a physiologic network^1,3^. An individual SA-agent:

1. Has sensors, with inputs receiving signals in the form of sound, vision, taste, tactile, or smell from its environment. The source of information can be from other SA-agents or its payoff, which SA-agent uses in the decision-making process or as feedback for reinforcing the corresponding *P*s, respectively.
2. Has a way of making decisions based on what it learned from past experiences (its model of the world) and received information. Decision-making is SA-agent’s way of changing its environment, such as moving (legs and hands) or sharing information (communication).
3. Has a way of updating its set of *P*s (its model of the world) based on the feedback it receives.
4. Has an incentive to improve its payoff/performance.

**Figure 1:**
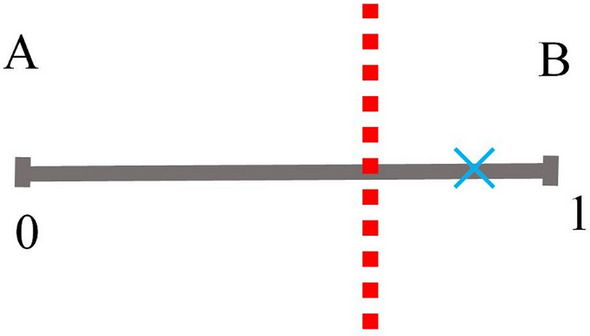
Schematics of the decision mechanism of SA-Agent for binary decision making. “*A*” and “*B*” represent the choices, and the red dotted line shows the position of the moving threshold, splitting the interval into *P* and 1 − *P*. The blue cross shows the random number generated at a given trial.

To make a decision at a given trial, the SA-agent generates a random number from a uniform distribution [0 1] that would fall in one of the intervals, which is to say, collapses the *P*s to a decision. For example, if the blue cross illustrated in Fig. 1 shows the value of the random number generated by the SA-agent, then the decision of the SA-agent in this trial is ’*B*.’ The random number that the SA-agent uses in each decision-making trial prevents the dynamics from being deterministic/fragile and helps the SA-agent sustain exploration. For example, if the value of *P* for a choice is small but non-zero, the SA-agent may still select its corresponding choice.

The decision-making process of a SA-agent (2 above) relies on adaptive *P*s. In the case of binary choice, *P* and 1 − *P* represent the propensity of the two complementary choices. In Fig. 1, we demonstrated this using a moving threshold which divides the interval [0 1] into two sections, each corresponding to a choice (*A* or *B*).

Note that if there were *d* choices for a SA-agent to pick from, then *d* − 1 thresholds are needed, and the intervals between those thresholds correspond to the *P*s of the choices. Also, the SA-agent might have different choices which should be taken consecutively. For example, a SA-agent in^8^ must first choose another SA-agent with whom to play the PD game, then making a choice between “*C*" and “*D*”, and finally choose to trust or not to trust the decision of the other SA-agent. After decision-making, the SA-agent receives a payoff and uses that payoff as feedback to update the position of the corresponding thresholds (values of the *P*s) that contributed to the final choice (reinforcing the *P*s). The process of updating the *P*s (3 above) requires the SA-agent to:

1. Save the position of the threshold(s) and the payoff of the current and previous trial.
2. Use Eq. 2 to reinforce (increase the *P* of the successful decision(s)).

Note that a SA-agent’s model of the world, represented in complex networks of *P*s, is based on fuzzy logic and forms based on selfishness, which makes it interpretable. In this spirit we note further that the continuous reinforcement of the *P*s allows each SA-agent to continuously adapt its model of the world and, with that, anticipate/engineer the next state of its environment.

### Multiple SA-agents

In our model two groups of SA-agents interact with one another on a 2D plane with periodic boundary conditions. SA-agents of group 1(2) can move with velocities of magnitude *V*_1_(*V*_2_) and can detect the velocity and position of other SA-agents if those are in their vision radius *r*_1_(*r*_2_). If a SA-agent of group 1(2) has some SA-agents of group 2(1) in its vision radius, it receives the payoffs of an anti-coordination game as follows: For a SA-agent in group 1, if *n* (> 0) is the number of SA-agents of group 2 in *r*_1_, it receives a payoff of − *n*. Otherwise, it receives 1. For a SA-agent in group 2, if *m* (> 0) is the number of SA-agents of group 1 in *r*_2_, it receives a payoff *m*. Otherwise, it receives the payoff − 1.

**Figure 2:**
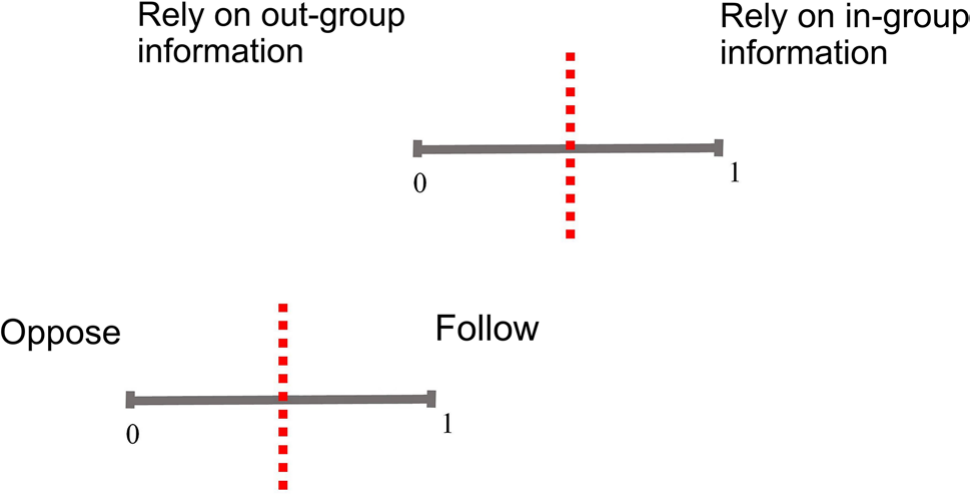
Schematics of the two-level binary decision mechanism of SA-Agent used in this work. Each SA-agent makes a decision based on the *P*s of the corresponding choice and the random number generated in the trial. At the first level, the SA-agent decides to either use the information of its neighboring in-group or out-group SA agents (Reliance, *R*(*t*) threshold). If its first decision is to use the in-group information it selects its next direction as of the average of those SA-agents. On the other hand, if it decides to use the information of the out-group, it uses the second level of decision making, i.e., it evaluates the next position of those SA-agents and decides either to move away (oppose) or go toward (follow) that point (oppose-follow, *OF*(*t*) threshold).

The decision process of the SA-agent used in this study is shown in Fig. 2. Each SA-agent can use the information (the velocity and position) of in-group or out-group SA-agents in its vision radius. The decision on which information to use is adaptive for each agent. So, SA-agents have a reliance (*R*(*t*)) of *P* values, which is the first level of decision making. If the SA-agent, based on its *R*(*t*), decides to use the in-group information, it picks its next direction to be the average of those SA-agents. If there are no in-group SA-agents in its neighborhood, it keeps its previous direction. On the other hand, if the SA-agent decides to use the information of out-group SA-agents, it uses the second level of decision making in which it anticipates the position of those SA-agents and has to decide to pick a direction away from or toward that position based on its oppose-follow (*OF*(*t*)) *P* values. Again, if no out-group SA-agents are in its neighborhood, it keeps its previous direction.

In our simulations, for simplicity, we studied cases where the number of SA-agents of each group is the same *N* = *N*_1_ = *N*_2_, the magnitude of their velocity (*V*_1_ = *V*_1_ = 0*.*01), and their vision radius (*r*_1_ = *r*_2_ = 0*.*15) are equal. The length of the 2D plane, with periodic boundary conditions, is 1. The change in the *P*s, quantified by ∆_*i,t*_, is a function of the last two payoffs of agent *i*^8^:

5 $$\Delta_{i,t} = \chi \frac{{\Pi_{i} (t) - \Pi_{i} (t - 1)}}{{\left| {\Pi_{i} (t)} \right| + \left| {\Pi_{i} (t - 1)} \right|}},$$

where *χ* = 0*.*2 is a positive number that represents the sensitivity of the SA-agent to the feedback it receives from its environment. The quantity Π_*i*_(*t*) is the payoff to the agent *i* at time *t*. If the value of *P* becomes less than 0 or more than 1, it is set back to 0 and 1, respectively. We note that these choices of parameters don’t change the main conclusions of this paper.

### Modifying the DEA

**Figure 3:**
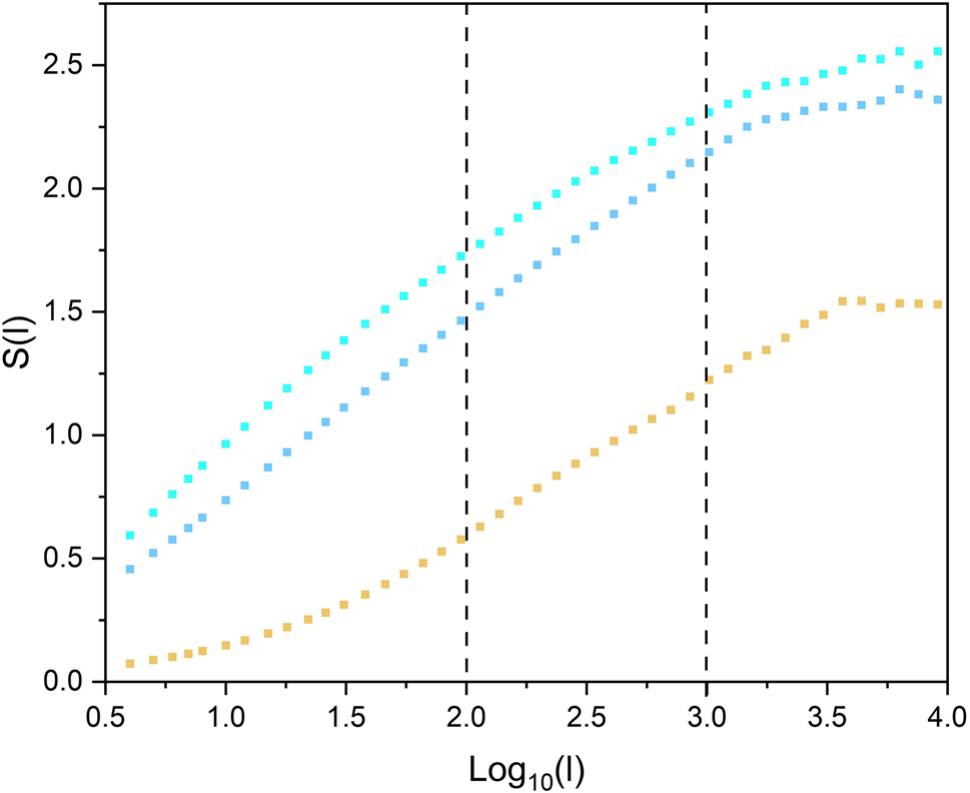
The DEA graph of three slices (each with length of 3 × 10^4^) of the averaged *R*(*t*) (blue), the averaged *OF*(*t*) (cyan), and the order parameter *O*(*t*) (yellow) of SA agents of group 1. The corresponding slopes of the linear sections are *δ* = 0.66, 0.71, and 0.62, respectively. *N* = 20. Stripe size = 0.001.

Shortly after the introduction of the DEA formalism^19^ it was determined that empirical data contained not just CEs as had been originally assumed but it was instead at least a mixture of CE time series and RE time series, the latter containing non-CE memory. Thus, it was necessary to generalize the DEA to filter out this source of correlated noise leading to a number of versions of modified DEA^24,46^. In this work we used the modified DEA introduced in^1^ where the input time series (such as ECG) changed to a discrete time series of events, defined using stripes, and DEA run to measure the scaling of diffusion process generated with those events. This modification lets us measure the scaling of short time series which is essential for detecting CS. Figure 3 shows the entropy versus *log*_10_(*t*) graph resulting from a modified DEA on three slices of time series of the averaged *R*(*t*) (i.e., ensemble average over the threshold time series of N SA-agents of the same group), averaged *OF*(*t*), and order parameter *O*(*t*). The slopes of the linear sections of the curves measure their scaling *δ*.

### Renewal experiment for the events of an SA-agent’s trajectory

**Figure 4:**
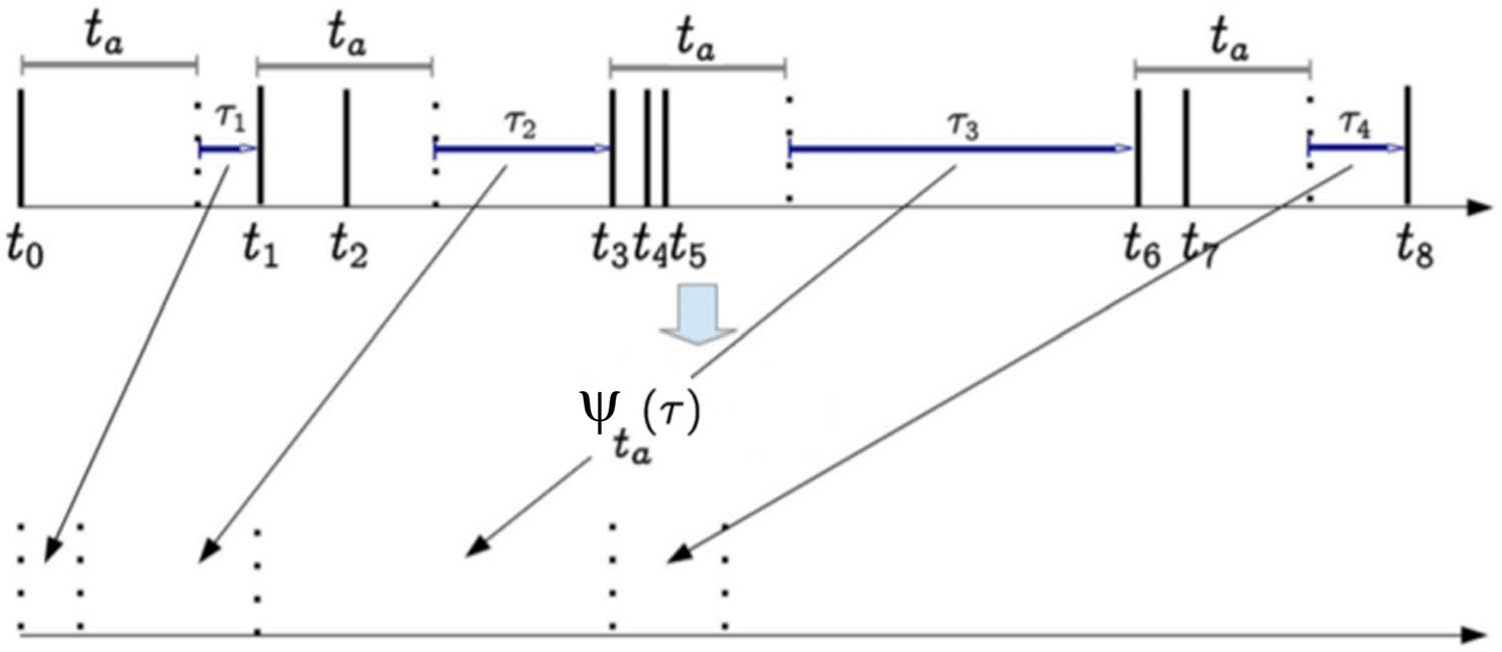
Schematics for the aging experiment. The time interval *τ*_*i*_, extracted using stripes aged by *t*_*a*_. From^14^ with permission.

To check whether or not the events extracted using stripes are renewal (i.e. consecutive events are statistically independent of one another), we use the renewal experiment (RE) introduced in^46^. The first step in the RE is aging the events with a given time *t*_*a*_. For aging, as shown in Fig. 4, we start from the events, and the time distance to the next event is the aged event. By collecting the aged time intervals, we can evaluate their waiting-time PDF. Note that aging influences small events more than large ones, and after normalization, the weight of short time intervals decreases while the weight of the large time intervals increases. Also, note that aging removes some of the short time events.

To check that the extracted events are renewal, i.e., independent consecutive events, we first shuffle the original τ_*i*_ and then age them and finally evaluate the waiting-time PDF of the resulting time intervals. If the waiting-time PDF of the two cases of aged and shuffled-aged are the same, it shows that there is no correlation between the events (i.e. they are CEs).

### Detrended fluctuation analysis of the EI

Detrended Fluctuation Analysis^47,48^ DFA has been used to study time series such as reaction time fluctuations^49^ and heart rate variability^50^. We used DFA to measure the scaling of the averaged *R*(*t*), averaged *OF*(*t*), and *O*(*t*) time series, as an alternative measure of complexity, and to compare its results with that of the DEA.

## Results

We analyzed the change of the complexity, measured by a modified DEA, of the averaged reliance *R*(*t*), averaged oppose-follow *OF*(*t*), and also of the order parameter *O*(*t*) of the two groups of SA-agents playing the anti-coordination game. The averaged *R*(*t*) and *OF*(*t*) time series are characteristics of the internal interactions of the system while the *O*(*t*) time series is a measure of the global behavior of the SA-agents.

**Figure 5:**
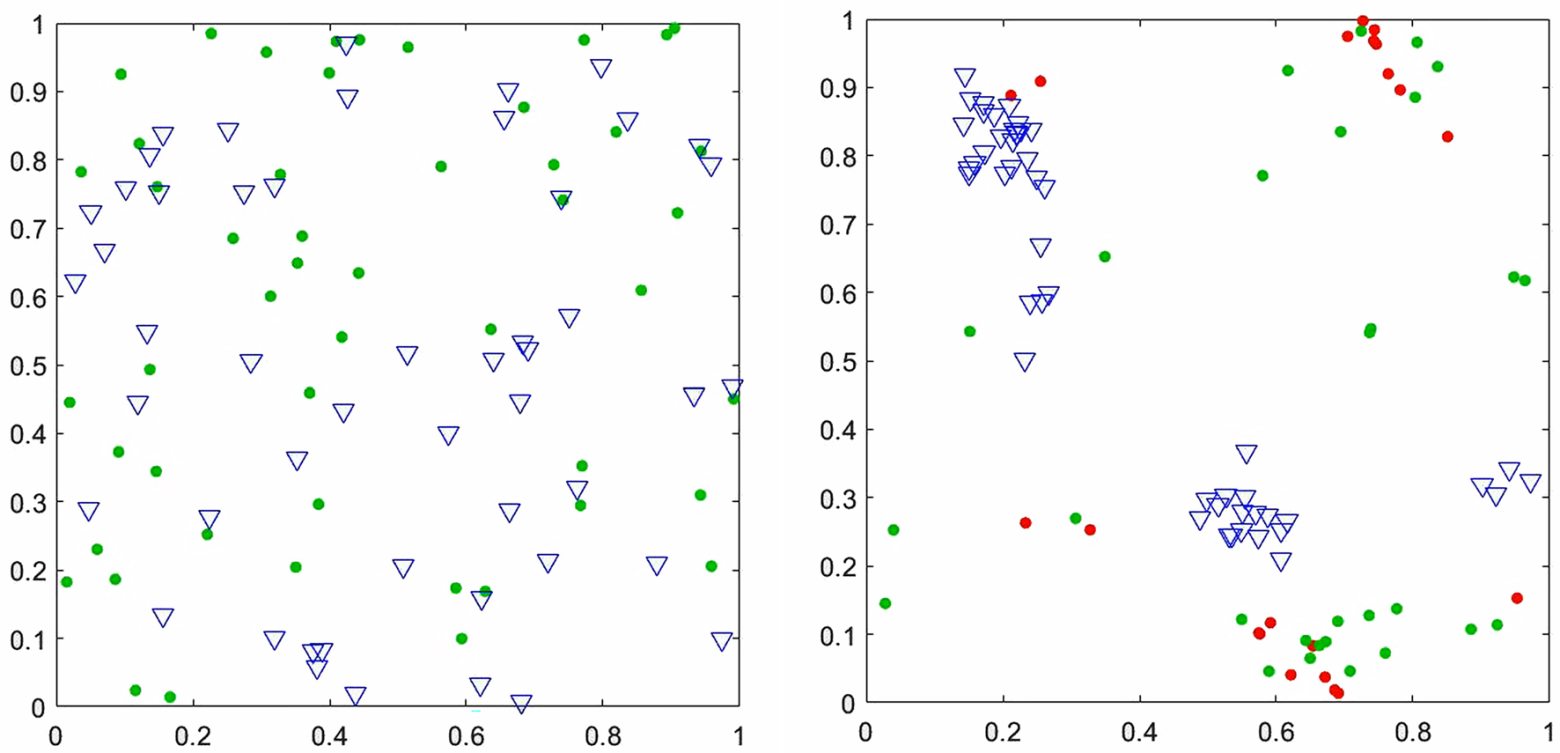
Snapshots from the position of the *N* = 50 SA-agents of group 1 (circles) and *N* = 50 SA-agents of group 2 (triangles) playing the anti-coordination game as described in the text. The left snapshot is taken at trial *t* = 1 while the snapshot on the right is taken after *t* = 10^4^ trials. The SA-agents of group 1 with *R*(*t*) < 0*.*25 are shown with red circles (representing the SA-agents with high propensity (*P* >  = 0*.*75) to rely on in-group information) and otherwise as green circles (for clarity, we haven’t distinguished the SA-gents of group 2 in this way.)

Figure 5 shows two snapshots of the configuration of SA-agents. At the beginning of the simulation (left panel), where the *N* = 50 SA-agents of two groups distinguished by their distinctive shapes were randomly distributed over the spatial checkerboard with equally random orientation. Subsequently, after interacting for *t* = 10^4^ trials (right panel), swarms of SA-agents of the same groups are formed. The change in the coloring denotes different parameter values, i.e., how they feel about what to do next.

**Figure 6:**
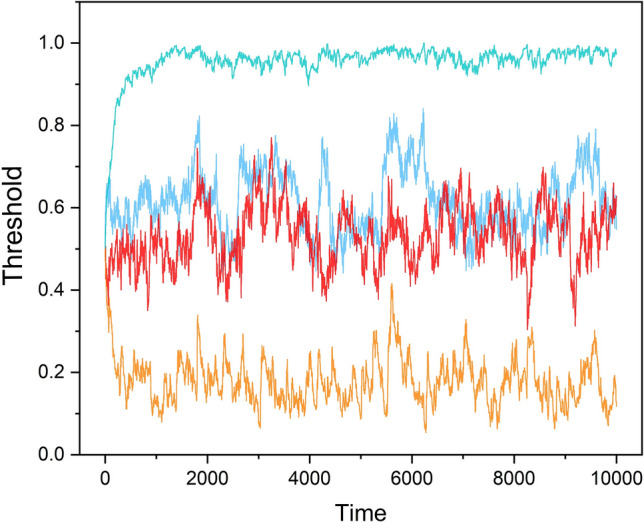
The evolution of the averaged *R*(*t*) (blue and red curve respectively for group 1 and 2) and averaged *OF*(*t*) (cyan and orange curve respectively for group 1 and 2). *N* = 20.

Figure 6 depicts the evolution of the averaged *R*(*t*) and *OF*(*t*) time series of the two groups, each with *N* = 20 SA-agents. The averaged *R*(*t*)s converge on the central region very quickly (blue and red curves), whereas the averaged *OF*(*t*)s go to opposing extremes just as quickly (cyan and orange curves).

**Figure 7:**
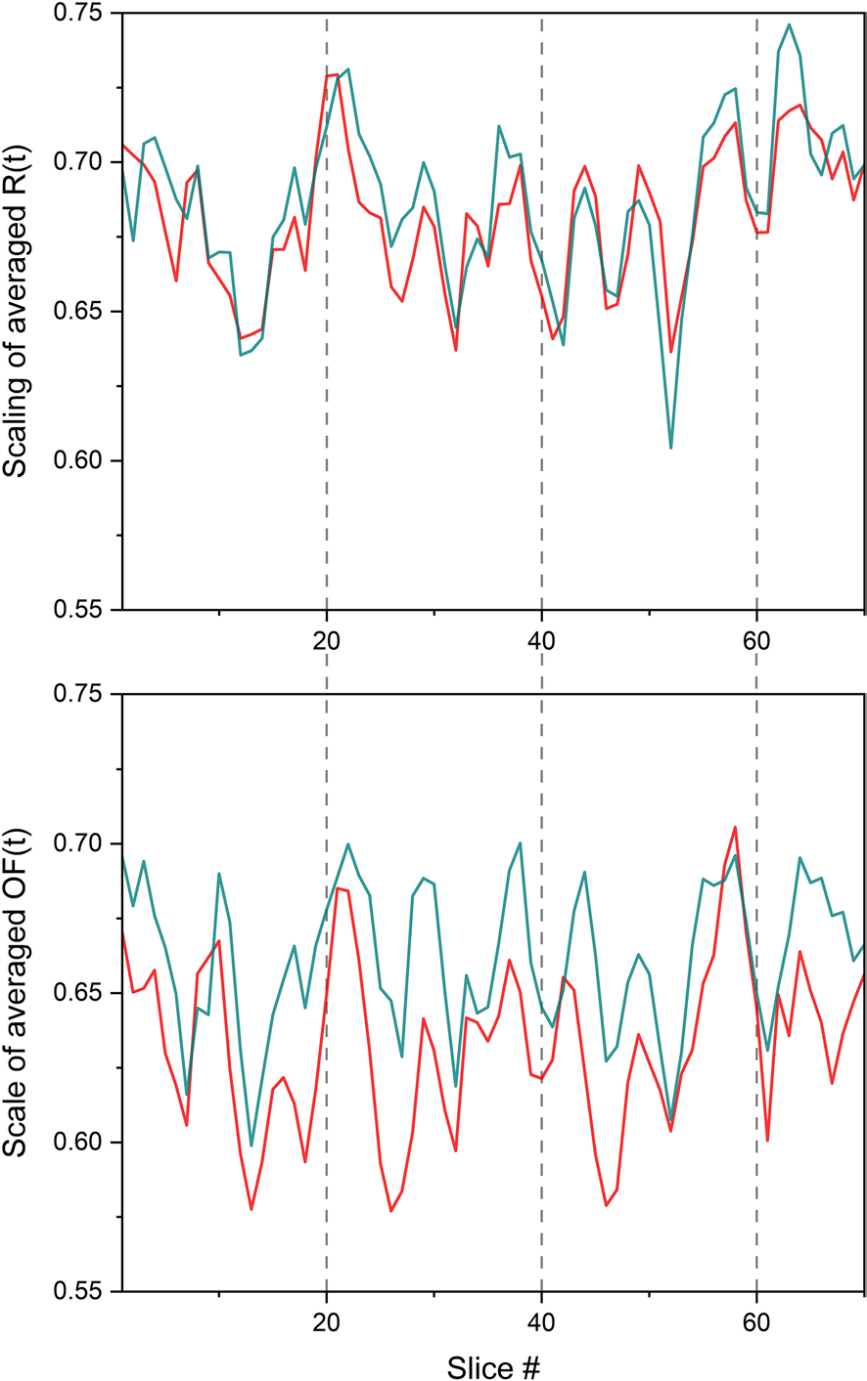
The scaling time series of averaged *R*(*t*) (top panel) and averaged *OF*(*t*) (bottom panel) thresholds time series of the group 1 (cyan) and group 2 (red) SA-agents. *N* = 20. The scalings are evaluated using a modified DEA (stripe size = 0.001) on slices with size 3 × 10^4^, moving by 1 × 10^4^ data points, where the length of the whole time series was 10^6^. The vertical dashed lines are to show the synchrony between the top and bottom scaling time series. The cross-correlations of the scaling time series are given in Table 1.

Figure 7 shows the scaling indices, resulting from applying a modified DEA to the time series of averaged *R*(*t*) and averaged *OF*(*t*) of SA-agents of groups 1 and 2. The scaling index time series was measured using slices of 3 × 10^4^ data points (trials) overlapped by 1 × 10^4^ data points. The resulting scalings are found to be time dependent and consequently to be multifractal.

**Figure 8:**
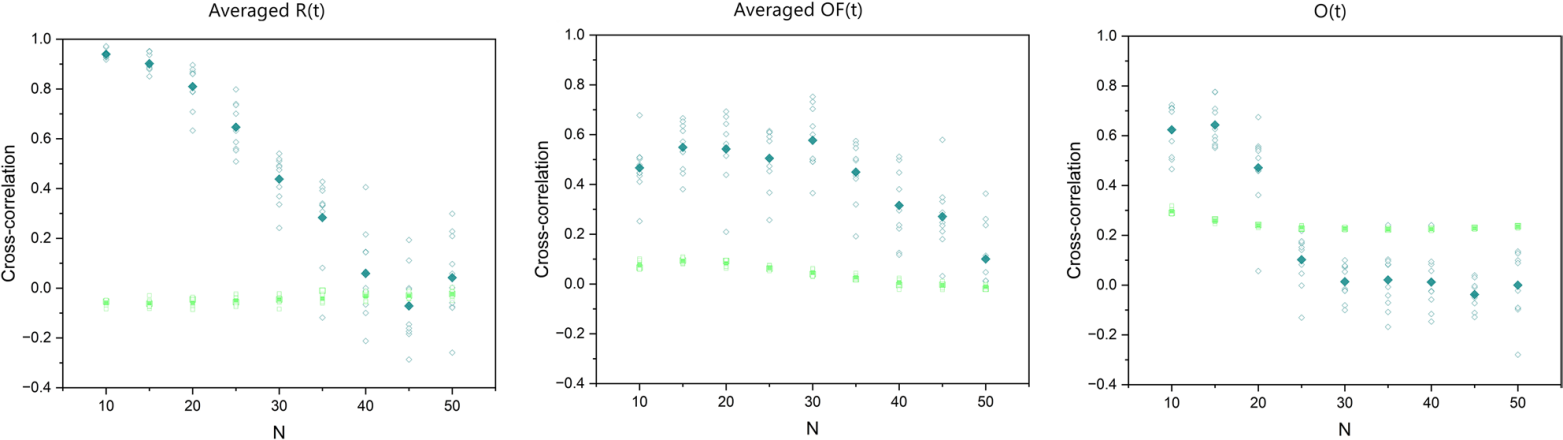
In the panels, from left to right, the cyan diamonds show the cross-correlation between the scaling time series evaluated using a modified DEA (with stripe size = 0.001) on averaged *R*(*t*), averaged *OF*(*t*), and on *O*(*t*) time series of the two groups, versus the number of SA-agents of each group *N*. The green squares show the average cross-correlation between the corresponding slices of data that were used for a modified DEA. The filled symbols show the mean values of the cross-correlations of the ten simulations done for each size *N*.

The cyan diamond symbols in the panels of Fig. 8, from left to right, show the cross-correlation between the time series of the scaling indices (similar to the ones depicted in Fig. 7), evaluated via a modified DEA on the averaged *R*(*t*), averaged *OF*(*t*), and the order parameter *O*(*t*) time series of the two groups of SA-agents for different group sizes *N*, respectively. The green squares are the cross-correlations between the corresponding time series (i.e., the time series sliced as was done for a modified DEA and their cross-correlations averaged). Ten simulations were done for each *N*. While the cross-correlations between the time series remain very low and insensitive to change of *N*, the cross-correlations between the scaling time series are high and measure the intelligence of the system.

The cross-correlation between the scaling time series of averaged *R*(*t*), averaged *OF*(*t*), and *O*(*t*), for *N* = 20, are given in Table 1.

**Table 1:** Cross-correlation between the scaling time series of the order parameter (*O*(*t*), averaged *R*(*t*), and averaged *OF*(*t*) time series of both groups (G1 and G2).

O-G1	O-G2	R-G1	R-G2	OF-G1	OF-G2
O-G1					
O-G2	0.6114				
R-G1	0.6247	0.5903			
R-G2	0.5560	0.5263	0.8787		
OF-G1	0.4729	0.3738	0.7569	0.6781	
OF-G2	0.4231	0.3811	0.6684	0.6765	0.6944

*N* = 20. *p*-values < 0.001.

**Figure 9:**
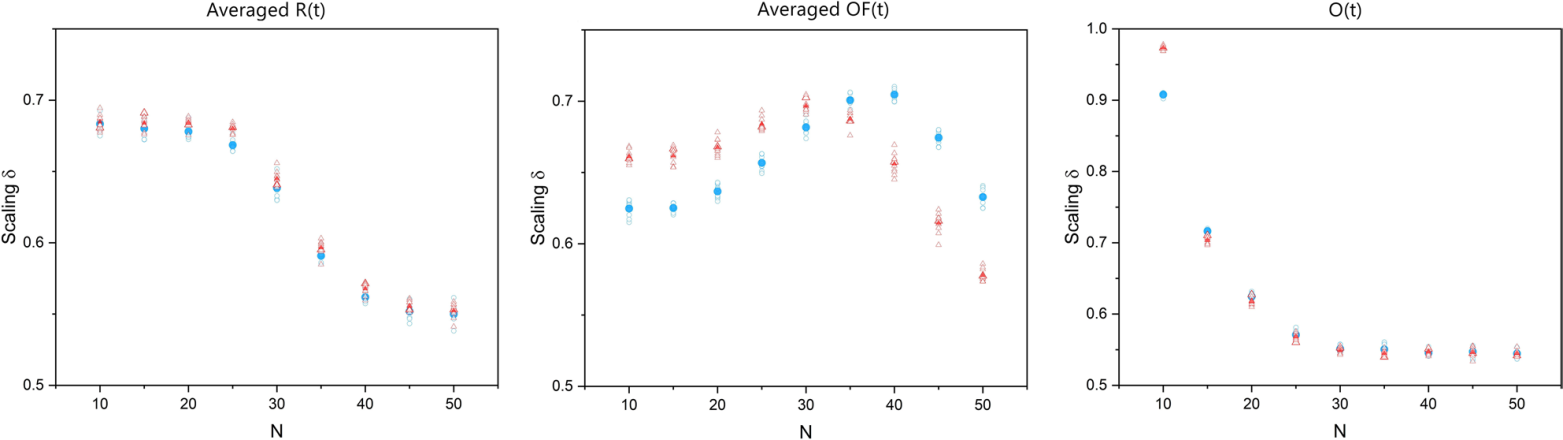
The panels, from left to right, show the average of the scaling time series, evaluated using a modified DEA (with stripe size = 0.001) on the averaged *R*(*t*), averaged *OF*(*t*), and *O*(*t*), versus the number of SA-agents of each group *N*. The blue circles and red triangles represent groups 1 and 2, respectively. The filled symbols show the mean values of the ten simulations done for each size.

Figure 9 shows the average of scaling time series versus SA-agent group size *N*. In the left panel of the figure, there is a plateau where the average scaling of the averaged *R*(*t*) remains constant for both groups and then starts decreasing at *N* = 20. In the middle panel there is a maximum value for the average scaling of the averaged *OF*(*t*) which occurs in different *N* for each group. In the right panel of the figure the average scaling of the *O*(*t*) dramatically decreases with increasing group size *N* for both groups.

**Figure 10:**
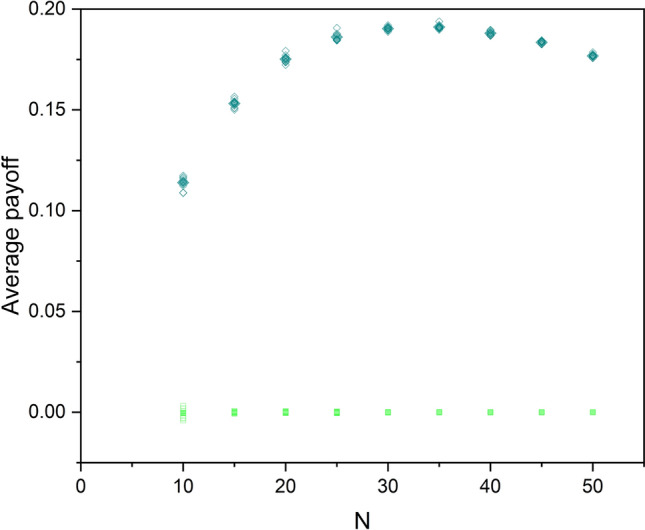
The cyan diamonds show the time average of the total payoff of the networks for different group size *N*. The green squares show the time average of the total payoff of the same systems in the absence of interaction between the SA-agents. The filled symbols show the mean values of the ten simulations done for each size.

The cyan diamonds in Fig. 10 show the average (ensemble averages and time averages) payoff of the overall system for each group size *N*. The average payoff increases by increasing *N* and reaches a maximum of about 0.2 and after that gradually decreases with increasing group size *N* for both groups. The green squares show the average payoff of the whole system if there were no interaction among the SA-agents. In this case, the average payoff remains very close to zero.

**Figure 11:**
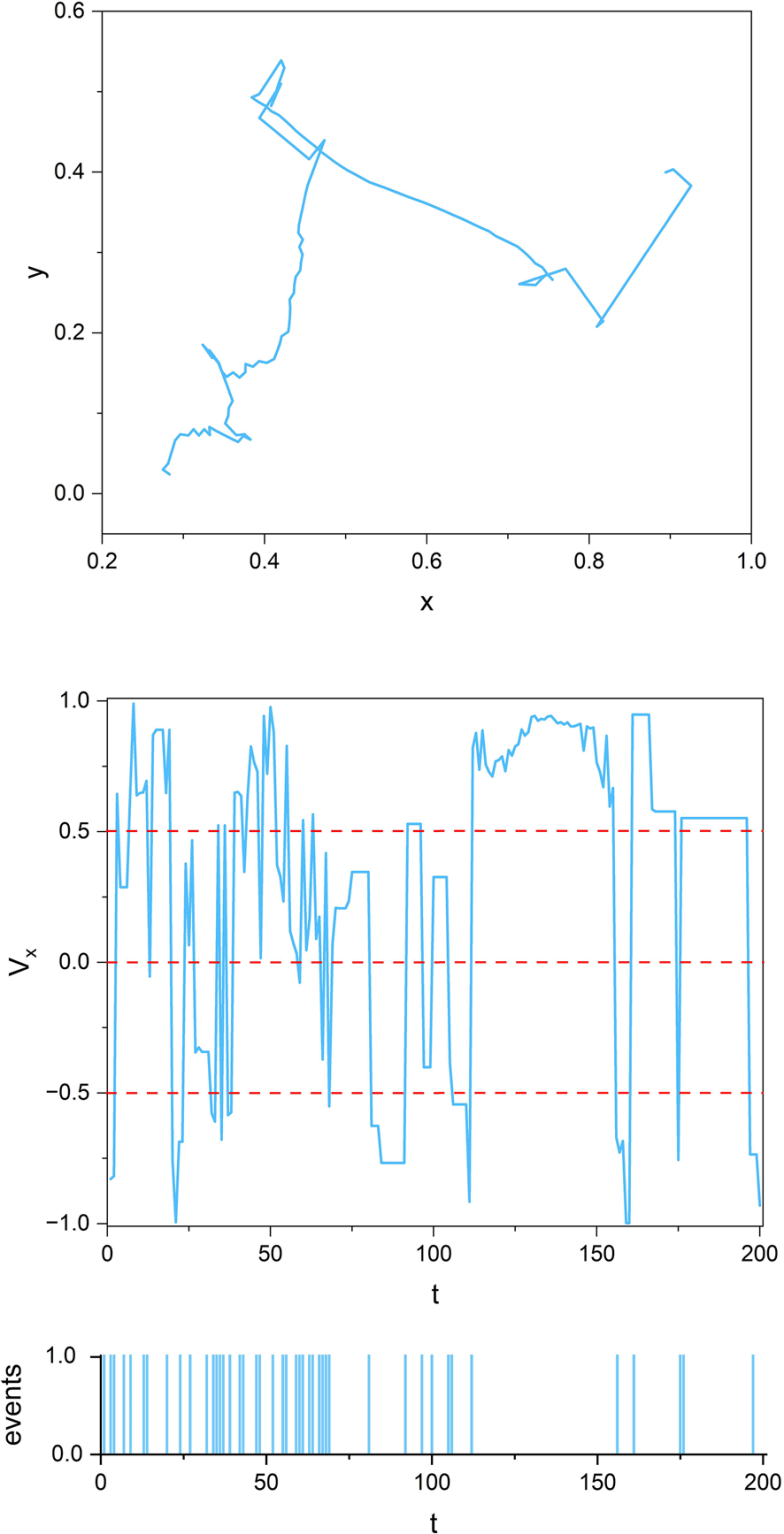
Schematics of the use of stripes in extracting events as a discrete/binary representation of the data. Top panel: part of the 2D trajectory of one SA-agent of group 1 (out of 19 group 1 and 20 group 2 interacting SA-agents). Middle panel: the time series of the velocity of the agent (top panel) projected onto the X-axis, *V*_*x*_. The horizontal red dotted lines depict four stripes, and every time the *V*_*x*_ time series passes across one stripe region into another, it is marked as an event (the blue bars in the bottom panel.

The top panel of Fig. 11 shows the trajectory of a single SA-agent of group 1 where it is interacting with the other 19 in-group and 20 out-group SA-agents (*N* = 20). The advantage of studying a single SA-agent’s trajectory is that the events are clearly marked as the discrete times the SA-agent changes its direction in the one-dimensional projection of that trajectory (middle panel). The projection of the 2D trajectory of the SA-agent onto the X-axis assists us in illustrating how in processing empirical datasets stripes extract events (bottom panel).

**Figure 12:**
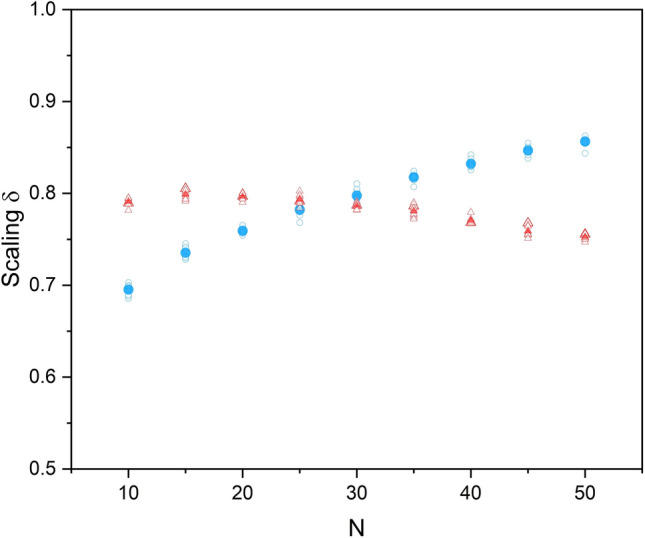
The average scaling of a single SA-agent’s trajectory for different *N*. The blue circles and red triangles represent groups 1 and 2, respectively. The filled symbols show the mean values of the ten simulations done for each *N*.

Figure 12 shows the average scaling of single trajectories for different *N*. The values of the scaling parameter all fall in the interval (0.65, 0.9).

**Figure 13:**
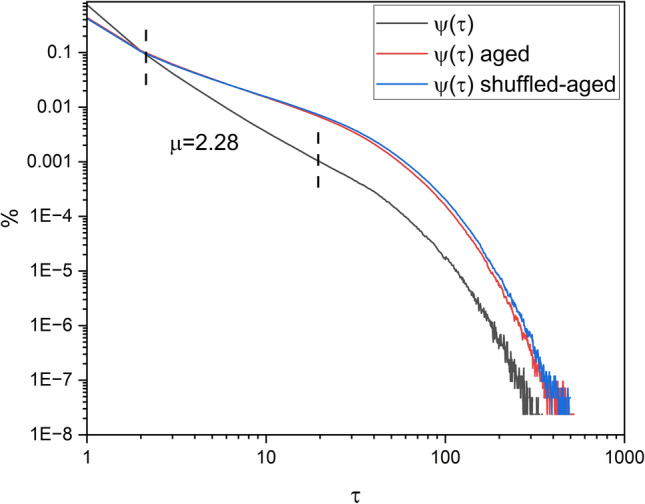
Renewal test for the events extracted using stripes from the velocity of the projection of the 2D trajectory of a single SA-agent from group 1, onto the x-axis *V*_*x*_(*t*). The black curve shows the waiting-time PDF of the extracted events, with IPL of *µ* = 2*.*28. The red and blue curves are the waiting-time PDF of the same events after being aged and being shuffled-aged, respectively. *N* = 20. Stripe size = 0.01, *t*_*a*_ = 100, length of the time series = 10^7^ trials.

Figure 13 shows the results of the renewal experiment done on the events extracted using stripes from the velocity of the projection of the 2D trajectory of a single SA-agent from group 1. The curves show the waiting-time PDF of the events, extracted using stripes, before aging, aged, and shuffled-aged.

**Figure 14:**
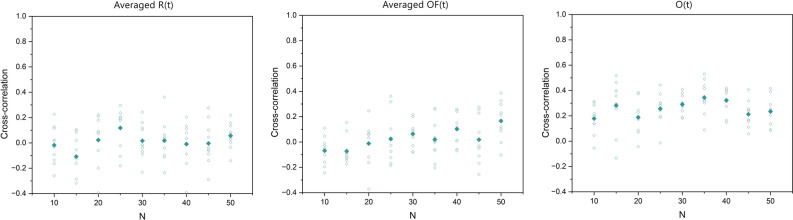
In the panels, from left to right, the cyan diamonds show the cross-correlation between the scaling time series evaluated using Detrended Fluctuation Analysis (DFA) on averaged *R*(*t*), averaged *OF*(*t*), and *O*(*t*) time series of the two groups, versus the number of SA-agents of each group *N*. The filled symbols show the mean values of the cross-correlations of the ten simulations done for each size *N*.

Figure 14 shows the cross-correlation between the scaling time series evaluated via DFA (rather than DEA) on the averaged *R*(*t*), averaged *OF*(*t*), and *O*(*t*) time series for different size *N*. Figure 14 shows the average of the DFA scaling time series for different size *N*.

## Discussion

In this section we provide some brief comments to provide insight into the calculations done and depicted in Figs. 4, 5, 6, 7, 8, 9, 10 and 11 that facilitate our understanding of the algorithmic reasoning leading to emergent intelligence (EI).

Figure 5 depicts that swarms emerge among SA-agents as they learn from their experience that adaptively changing their *R*(*t*) and *OF*(*t*) thresholds is beneficial. Note that without interaction between the SA-agents, the payoff of the overall system is close to zero for all values of *N*. On the other hand, for the interacting SA-agents, the sum of the payoff is a positive value (Fig. 10.) The time series of the averaged *R*(*t*) and averaged *OF*(*t*) of the SA-agents represents the interactions in the network while the *O*(*t*) shows the organization’s output. We analyzed the complexity of these time series for both groups of SA-agents using a modified DEA.

We stress that the ensemble averages of these two time series depicted in Fig. 6 constitute the internal decision making processes of the SA-agents, so we are witnessing the ’cognition’ of the SA-agents. Initially *R*(1) = 0*.*5 and *OF*(1) = 0*.*5, but over time, the SA-agents learn to adaptively use the information of the in-group and out-group SA-agents and, if they decide to use out-group information, the SA-agents of group 1 learn to migrate away (cyan curve), while the SA-agents of group 2 learned to migrate towards (orange curve) the anticipated position of the SA-agents of the other group.

It is evident from Fig. 7 that scaling parameters for the averaged *R*(*t*) of the two groups are in synchrony, as are the scaling parameters for the averaged *OF*(*t*). Thus, the scaling behavior of both time series characterizing the SA-agents ’cognition’ are multifractal manifesting CS with quasi-periodic scaling. Although not having any mechanism in common with the interacting triad of ONs involving the heart, lungs, and brain, the theoretical social SA-agent model depicted in the figure manifests the same CS phenomenon first observed in processing the empirical data from the interacting triad^1,3^.

A third CS is suggested by the vertical bars in Fig. 7 introduced to aid the eye in comparing the quasi-periodic variability of scaling time series of averaged *R*(*t*) and averaged *OF*(*t*) time series. Note that the CS of the averaged *R*(*t*) time series appears to be much greater than that of the averaged *OF*(*t*) time series. The potential significance of this difference ought to be explored and we plan to do so in subsequent studies focusing on the parameters of the model.

In the left panel of Fig. 8 the cross-correlation between the scaling time series of averaged *R*(*t*) of the two groups is > 0*.*95 for *N* = 10 and is diminished with increasing group size *N*. In the middle panel of this figure the cross-correlation between the scaling time series of averaged *OF*(*t*) of the two group increases in value non-monotonically until reaching a maximum of about 0.6 at *N* = 30 and then non-monotonically decreases with increasing group size *N*. In the right panel of this figure the cross-correlation between the scaling time series of the order parameter *O*(*t*) of the two groups is similar to that of the averaged *R*(*t*) but lower in intensity and diminishes at smaller *N*. Unlike the first two panels, which depict the influences on the decision-making process, the order parameter depicts the results of the decision-making on the average behavior of the SA-agent.

The results show that these time series have anomalous scaling in the range of (0.5, 1). More importantly, our results show that the scaling of these time series varies in time in a way that are in synchrony with one another (see Figs. 7 and 8, and Table 1). This synchrony phenomenon was recently found in the time series of EEG, ECG, and Respiratory data^1,3^, wherein the name Complexity Synchronization (CS) was coined. As Fig. 8 shows, CS can distinguish between different systems of size *N* while the typical cross-correlation between the time series is very low and is not sensitive to changes of *N*.

The panels of Fig. 9 show that the average of the scaling time series of these time series also depends on *N*. These results suggest that CS is a general property of mutual adaptive environments that can be used to quantify EI at both the levels of the average of the time series scaling index and the magnitude of their cross-correlations.

The social model we used for CS in EI is a MABM utilizing SA-agents. Rather than discussing the details of the EI using this model for different parameters, we used it to show that CS is a natural outcome of self-organization. This social model encourages future theoretical work on CS in a totally different context from that in which it was discovered. Note that our social MABM should not be confused with models where the dynamics of the system rely on a macroscopic control parameter that must be set by the experimenter. For example, in the Vicsek model^36^, the uncertainty in measuring the average velocity by the agent is introduced in the model as noise, which plays the role of a control parameter, and in^51^, the selection strength *β* is the macroscopic control parameter. In our social MABM, there is no control parameter; instead, the other SA-agents constitute the environment of the SA-agent of interest and they replace the role of control with a bottom-up self-organizing process originating from the self-interest of the SA-agents.

### Emergence of CEs

Viswanathan et al. in^52^ studied the foraging behavior of a prototypical wandering albatross and found an IPL distribution of flight time intervals with *µ* = 2, indicating its flight path is a Lévy process. They interpreted the existence of such temporal scale invariance to be a consequence of a possible scale-invariant spatial distribution of food resources for the albatross. This suggested that we focus our analysis on the behavior of a single SA-agent and because of its interactions with other SA-agents, its dynamics constitute a Lévy Walk.

The middle panel of Fig. 11 shows the velocity of the same SA-agent projected onto the X-direction *V*_*x*_(*t*), which is indeed the projection of the 2D trajectory shown in the top panel. The three red-dotted lines in the middle panel divide it into four stripe regions, and every time the *V*_*x*_(*t*) passes from one stripe region to another we interpret that crossing as an event (marked as an event of positive unit amplitude), as depicted in the bottom panel. This creates a discrete/binary representation of the dynamics of the original time series. In a modified DEA, we use these randomly spaced events to create the steps for a diffusion process. Note that the bottom panel of this figure is equivalent to panel b of Fig. 2 in^1^. In the case of empirical data, it is often the average of many signals, which, using stripes, can recover events and use them to carry out the entropy analysis of the empirical diffusion process.

Figure 12 shows the average scaling *δ* of a single SA-agent’s trajectory from group 1 and group 2. The scalings indicate anomalous behavior, the first sign of the emergence of a Lévy Walk.

We also show, using the aging experiment^46^, that the events are renewal, specifying the emergence of crucial events (CEs) with statistics similar to those of a Lévy Walk.

Figure 13 shows the waiting-time PDF of the events, extracted using stripes, for the *V*_*x*_(*t*) before aging, aged, and shuffled-aged. There is IPL in the waiting-time PDF of all three cases. Also, the waiting-time PDF of the aged and shuffled-aged events are the same, which shows the events are renewal, confirming that the dynamics of the single SA-agent is ruled by the renewal process (CEs). Note that the evaluated IPL of the waiting-time PDF of the extracted events *µ* in Fig. 13 is related to *δ* in Fig. 12 by equation *µ* = 1 + 1*/δ*.

We highlight that, however, the scaling in our model changes over time rather than being fixed, as it is in the case of a simple Lévy Walk. This change from a constant to a time-dependent scaling parameter depicts the transition from a monofractal to a multifractal process. These results show that in the social MABM a Lévy PDF is an emergent property of mutual interaction between the two competing groups of SA-agents.

### DEA versus DFA

**Figure 15:**
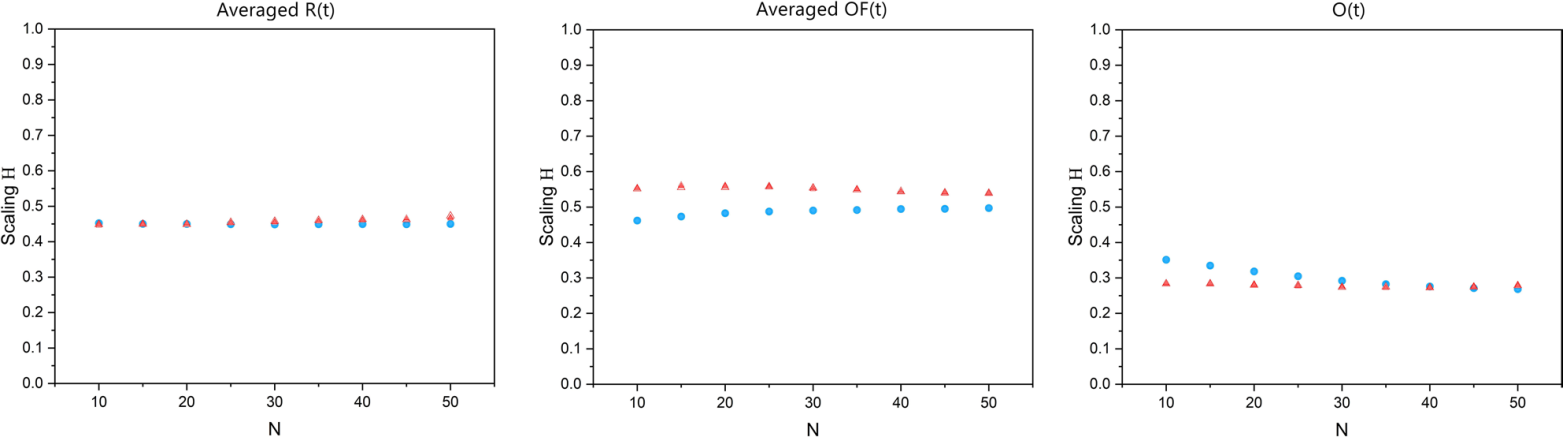
The panels, from left to right, show the average of the scaling time series, evaluated using Detrended Fluctuation Analysis (DFA) MDEA on the averaged *R*(*t*), averaged *OF*(*t*), and *O*(*t*), versus the number of SA-agents of each group *N*. The blue circles and red triangles represent groups 1 and 2, respectively. The filled symbols show the mean values of the ten simulations done for each size.

Figure 14 shows that, in contrast with Fig. 8, the DFA fails to reveal the existence of CS in the data and what that entails. This is because the DEA measures the dynamics of the system on a large time scale while DFA captures the dynamics of the local interactions. Figure 15, in comparison with Fig. 9, shows that the DFA scalings have less variability versus *N*, and while for the averaged *R*(*t*) and averaged *OF*(*t*) time series the DFA scalings show a process with almost no memory (H close to 0.5), the DFA scaling of the *O*(*t*) time series shows a scaling less than 0.5, indicating an anti-correlation in the signal.

These results show the advantage of a modified DEA used in this research in tracking the complexity of short time series.

## Conclusion

The implications of this research on complexity synchronization are dramatic and far-reaching in that the theoretical and analytical approaches that have proven useful in the understanding of this new phenomenon may generalize across a broad spectrum of questions from complex human neurophysiological ONs to complex social networks of humans, as well as into the realm of technology networks. We are pursuing a more comprehensive research plan to translate complexity science concepts, methods, and tools to seek self-organizing and operating principles valid across both biological and sociological networks.

The findings recorded herein strongly suggest that the EI of SA-agents is a candidate for modeling the interactions with humans for training/teaming/rehabilitation^25^. Also, it is worth emphasizing the difference between the present modeling strategy and that involving artificial intelligence (AI) by pointing out that the EI of SA-agents is based on fuzzy logic and self-interest in contrast to AI being based on learning from selected human datasets and is without self-interest. This makes EI interpretable for humans, while AI is considered a “black box” in most of the published literature. Note that there is no macroscopic control parameter in the EI of SA-agents with control being a bottom-up emergent property of the underlying dynamic process.

Little is known about how to observe, manage, and improve biological/artificial/ hybrid human–machine interacting networks. In previous work, we showed that by examining biological datasets at the level of their scaling indices, there is synchrony among their complexity indices. In particular, the investigation presented herein establishes that although the MABM social model apparently has no mechanism in common with that of the interacting triad of ONs involving the heart, lungs and brain, the theoretical social SA-agent model manifests what appears to be the same CS phenomenon first observed in processing the empirical data from the interacting triad of ONs^1,3^.

Thus, we conjecture that just as all linear dynamic systems that are periodic in time, whether their periodicity is a consequence of a spinning wheel, an equidistant set of points along a line, the sound of single frequency musical note, or any of a large number of other physical processes, can be described using a simple harmonic oscillator, so too can all complex dynamic networks having CS in time, whether the CS is a consequence of multiple interacting ONs or of interacting social groups of arbitrary size, can be described by their synchronously locked measures of complexity, those being their multifractal dimensions (MFDs).

The present work uses a relatively simple MABM social model incorporating SA-agents into the network dynamics to show that CS is a general property of mutually adaptive environments and that the emerging CS between SA-agents’ time series is compatible with the CS in human biological/behavioral data. The ensemble average multifractal dimension < *D*(*t*) > is herein given by < *D*(*t*) >  = 2 −  < *R*(*t*) > and therefore we found in the *Results* section that < *R*(*t*) > has a quasi-harmonic variability in the relatively narrow interval [1.26,1.4]. This synchronous behavior displayed by < *R*(*t*) > in Fig. 7 is not that different from the quasi-periodic scaling of the MFD behavior of < *OF*(*t*) > parameter depicted there as well.

We close with a hypothesis based on the observation that the fractal dimension of a time series generated by an isolated complex network plays a role analogous to that of the frequency in a simple linear system. Moreover, the singularity spectrum of a MFD time series generated by a complex network plays a role analogous to that of a frequency spectrum in a fractionally dynamic linear systems. The hypothesis goes as follows^53^:

Universal Law of MFD Synchrony (ULMFDS): All ONs are described by Crucial Event time series having fractal dimensions when isolated. When allowed to interact with one or more other ONs to form a network-of-ONs (NoONs) each fractal time series becomes a MFD time series and the only configuration for a healthy NoONs has complexity synchronization to stabilize its operation.

## Data Availability

The data used to produce the figures are available at https://drive.google.com/drive/folders/1uZPi_e1T4_ 3DHh8ZAT1bct300m1U2z0B?usp = sharing.

## Code availability

All the codes used to produce the results of this work are available at https://github.com/Korosh137/Complexity-Synchroni

## References

[1] Mahmoodi K, Kerick SE, Grigolini P, Franaszczuk PJ, West BJ (2023). Complexity synchronization: A measure of interaction between the brain, heart and lungs. Sci. Rep..

[2] Mahmoodi K, Kerick SE, Grigolini P, Franaszczuk PJ, West BJ (2023). Temporal complexity measure of reaction time series: Operational versus event time. Brain Behav..

[3] West BJ, Grigolini P, Kerick SE, Franaszczuk PJ, Mahmoodi K (2023). Complexity synchronization of organ networks. Entropy.

[4] Strogatz, S. H. Sync: How order emerges from chaos in the universe. *Nature, Dly. Life* (2003).

[5] Pikovsky A, Rosenblum M, Kurths J (2002). Synchronization: A universal concept in nonlinear science. Self.

[6] Weibel ER (2000). Symmorphosis, on Form and Function n Shaping Life.

[7] Feder J (1988). Multifractal measures. Fractals.

[8] Mahmoodi K, West BJ, Gonzalez C (2020). Selfish algorithm and emergence of collective intelligence. J. Complex Netw..

[9] Earl MG, Strogatz SH (2003). Synchronization in oscillator networks with delayed coupling: A stability criterion. Phys. Rev. E.

[10] West BJ, Mahmoodi K, Grigolini P (2019). Empirical Paradox, Complexity Thinking and Generating New Kinds of Knowledge.

[11] Couzin I, Krause J, Franks N, Levin S (2005). Effective leadership and decision making in animal groups on the move. Nature.

[12] Vicsek T, Zafeiris A (2012). Collective motion. Phys. Rep..

[13] Castellano C, Fortunato S, Loreto V (2009). Statistical physics of social dynamics. Rev. Mod. Phys..

[14] West BJ, Grigolini P (2021). Crucial Events: Why are Catastrophes Never Expected?.

[15] Mahmoodi K, West BJ, Grigolini P (2020). Complex periodicity and synchronization. Front. Physiol..

[16] Bak P (1996). How Nature Works: The Science of Self-Organized Criticality.

[17] Mahmoodi K, West BJ, Grigolini P (2018). Self-organized temporal criticality: Bottom-up resilience versus top-down vulnerability. Complexity.

[18] Allegrini P (2009). Spontaneous brain activity as a source of ideal 1/f noise. Phys. Rev. E.

[19] Allegrini P, Barbi M, Grigolini P, West BJ (1995). Dynamical model for DNA sequences. Phys. Rev. E.

[20] Feller W (1950). Introduction to Probability Theory and its Applications, 2 Volumes.

[21] Grigolini P, Aquino G, Bologna M, Lukovic M, West BJ (2009). A theory of 1/f noise in human cognition. Phys. A: Stat. Mech. its Appl..

[22] Goris R, Movshon J, Simoncelli E (2014). Partitioning neuronal variability. Nat. Neurosci.

[23] Cox D (1967). Renewal Theory.

[24] Allegrini P, Grigolini P, Hamilton P, Palatella P, Raffaelli G (2002). Memory beyond memory in heart beating, a sign of a healthy physiological condition. Phys. Rev. E.

[25] West BJ, Grigolini P, Bologna M (2023). Crucial Event Rehabilitation Therapy: Multifractal Medicine.

[26] Aquino G, Bologna M, West BJ, Grigolini P (2011). Transmission of information between complex systems: 1/f resonance. Phys. Rev. E.

[27] West B, Geneston E, Grigolini P (2008). Maximum information exchange between complex networks. Phys. Rep..

[28] Almurad ZM, Roume C, Delignières D (2017). Complexity matching in side-by-side walking. Hum. Mov. Sci..

[29] Almurad Z, Roume C, Blain H, Delignieres D (2018). Complexity matching: Restoroing the complexity of locomotion in older people therough arm-in-arm walking. Front. Physiol. Fract. Physiol..

[30] Grigolini P, Leddon D, Scafetta N (2002). Diffusion entropy and waiting time statistics of hard-x-ray solar flares. Phys. Rev. E.

[31] Scafetta N, Grigolini P, Imholt T, Roberts J, West BJ (2004). Solar turbulence in earth’s global and regional temperature anomalies. Phys. Rev. E.

[32] Mahmoodi K, West BJ, Grigolini P (2017). Self-organizing complex networks: individual versus global rules. Front. Physiol..

[33] Mahmoodi, K. & Grigolini, P. Imitation-induced criticality: Network reciprocity and psycho-logical reward. *arXiv preprint *http://arxiv.org/abs/1512.00100 (2015).

[34] Mahmoodi K, Grigolini P (2016). Evolutionary game theory and criticality. J. Phys. A: Math. Theor..

[35] Marinazzo D (2014). Information transfer and criticality in the ising model on the human connectome. PloS One.

[36] Vicsek T, Czirók A, Ben-Jacob E, Cohen I, Shochet O (1995). Novel type of phase transition in a system of self-driven particles. Phys. Rev. Lett..

[37] Mahmoodi, K. & Gonzalez, C. Emergence of collective cooperation and networks from selfish-trust and selfish-connections. in *CogSci*, 2254–2260 (2019).

[38] Majdandzic A (2014). Spontaneous recovery in dynamical networks. Nat. Phys..

[39] Martinez ND, Williams RJ (2023). From neworks to networking. Nat. Phys..

[40] Pirani, M. & Jafarpour, S. Network critical slowing down: Data-driven detection of critical transitions in nonlinear networks. http://arxiv.org/abs/2208.03881v1 (2022).

[41] Chialvo DR, Cannas SA, Grigera TS, Martin DA, Plenz D (2020). Controlling a complex system near its critical point via temporal correlations. Sci. Rep..

[42] Moraes JT, Trejo EJA, Camargo S, Ferreira SC, Chialvo DR (2023). Self-tuned criticality: Controlling a neuron near its bifurcation point via temporal correlations. Phys. Rev. E.

[43] Jelinek HF (2021). Diffusion entropy versus multiscale and renyi entropy to detect progression of autonomic neuropathy. Front. Physiol..

[44] Chachan, S. Emergent intelligence: A novel computational intelligence technique to solve problems. in *11th International Conference on Agents and Artificial Intelligence* (2019).

[45] Rzevski G, Skobelev P, Zhilyaev A (2022). Emergent intelligence in smart ecosystems: Conflicts resolution by reaching consensus in resource management. Mathematics.

[46] Allegrini P, Bari F, Grigolini P, Paradisi P (2007). Aging and renewal in sporatically modulated systems. Chaos Solit. Fract..

[47] Peng C-K (1994). On the mosaic organization of DNA sequences. Phys. Rev. E.

[48] Peng C-K, Havlin S, Stanley HE, Goldberger AL (1995). Quantification of scaling exponents and crossover phenomena in nonstationary heartbeat time series. Chaos: Interdiscip. J. Nonlinear Sci..

[49] Simola J, Zhigalov A, Morales-Muñoz I, Palva JM, Palva S (2017). Critical dynamics of endogenous fluctuations predict cognitive flexibility in the go/nogo task. Sci. Rep..

[50] Rosenberg AA, Weiser-Bitoun I, Billman GE, Yaniv Y (2020). Signatures of the autonomic nervous system and the heart’s pacemaker cells in canine electrocardiograms and their applications to humans. Sci. Rep..

[51] Kleshnina M, Hilbe C, Šimsa Š, Chatterjee K, Nowak MA (2023). The effect of environmental information on evolution of cooperation in stochastic games. Nat. Commun..

[52] Viswanathan GM (1996). Lévy flight search patterns of wandering albatrosses. Nature.

[53] West, B. J. Complexity synchronizationin living matter: A mini-review. *Undr Review*.

